# Supplementary material for: ­­­­­­­A type I IFN, prothrombotic hyperinflammatory neutrophil signature is distinct for COVID-19 ARDS­­­
Source: Wellcome Open Res. 2021 May 20;6:38. Originally published 2021 Feb 22. [Version 2] doi: 10.12688/wellcomeopenres.16584.2 (PMC8112464; doi:10.12688/wellcomeopenres.16584.2)
Supplement: Peer review history

# Peer review: Reviewer response for version 2

**DOI:** 10.21956/wellcomeopenres.18650.r43998

**Published:** 2021-06-25

**Authors:** Véronique Witko-Sarsat

**Affiliations:** 1 Institut National de la Santé et de la Recherche Médicale U1016, Université Paris-Descartes, Paris, France

With regard with the revised version of the article, I am fully satisfied. The authors have amended the manuscript to properly solve the issues that I have raised. In my opinion, this version of the manuscript could be accepted for indexing.

Is the work clearly and accurately presented and does it cite the current literature?

Yes

If applicable, is the statistical analysis and its interpretation appropriate?

Yes

Are all the source data underlying the results available to ensure full reproducibility?

Yes

Is the study design appropriate and is the work technically sound?

Yes

Are the conclusions drawn adequately supported by the results?

Partly

Are sufficient details of methods and analysis provided to allow replication by others?

Yes

Reviewer Expertise:

NA

I confirm that I have read this submission and believe that I have an appropriate level of expertise to confirm that it is of an acceptable scientific standard.

# Peer review: Reviewer response for version 2

**DOI:** 10.21956/wellcomeopenres.18650.r43997

**Published:** 2021-06-03

**Authors:** Attila Mocsai

**Affiliations:** 1 Department of Physiology, Semmelweis University School of Medicine, Budapest, Hungary

I thank the authors for their responses and for improving the manuscript. This is an important and timely piece of work. Open questions need to be addressed in future follow-up studies.

Is the work clearly and accurately presented and does it cite the current literature?

Partly

If applicable, is the statistical analysis and its interpretation appropriate?

Yes

Are all the source data underlying the results available to ensure full reproducibility?

Yes

Is the study design appropriate and is the work technically sound?

Yes

Are the conclusions drawn adequately supported by the results?

Yes

Are sufficient details of methods and analysis provided to allow replication by others?

Yes

Reviewer Expertise:

neutrophil biology, inflammation

I confirm that I have read this submission and believe that I have an appropriate level of expertise to confirm that it is of an acceptable scientific standard.

# Peer review: Reviewer response for version 1

**DOI:** 10.21956/wellcomeopenres.18276.r43487

**Published:** 2021-05-12

**Authors:** Daniel Irimia

**Affiliations:** 1 Department of Surgery, Center for Engineering in Medicine, Massachusetts General Hospital, Boston, MA, USA; 2 Shriners Burns Hospital, Galveston, TX, USA

This study explores the changes in neutrophils during ARDS and, more specifically, the differences in neutrophils between covid-ARDS and ARDS of other causes. The neutrophil changes are measured using several assays, including flow cytometry, liquid chromatography–mass spectrometry (LC-MS), immunochemistry, and biochemistry with a focus on metabolic changes. While exploratory in nature, the study generates a number of interesting hypotheses that would be tested in further studies. 

 A major limitation of the study is the small size of the patient cohort.  While the fraction of the covid positive patient who end with complications and death is relatively small, a larger number of subjects is needed in order to avoid any bias in the results.

 The presence of a larger number of low-density neutrophils (LDN) in covid-ARDS vs. non-covid-ARDS is interpreted as a stimulation of the myeloid production in the bone marrow that releases a larger than normal fraction of immature neutrophils. Further support to this hypothesis is provided by the observation that prednisone treatment reduces the proportion of LDN. Moreover, the expression of surface markers is also presented as being consistent with the presence of immature neutrophils in the circulation. Although interesting, these findings represent just one snap-shot in the evolution of the disease. A longitudinal study that observes the progression of the neutrophil subpopulation changes would be helpful in revealing how long after the SARS-CoV2 infection these changes can be detected and if the neutrophil changes can be measured before or after the ARDS is diagnosed.

 The study also finds that the neutrophils in circulation are more activated in covid than in non-covid ARDS patients. This finding is supported by proteomic data and a more detailed analysis points out the activation of IFN I pathways in covid-ARDS neutrophils, which is consistent with the viral origin of the condition. This finding is also supported by the immunochemistry of the neutrophil granules, and the analysis of metabolic changes. Overall, these findings are intriguing because they would suggest that other stimuli activating the neutrophils play a smaller role in changing the neutrophil phenotypes. A limitation of this analysis is the small number of samples analyzed.

 One of the most intriguing results reported in this study is that of the neutrophil-platelet aggregates. This finding may be potentially relevant to the higher rate of clotting observed in covid patients. However, this finding was not supported by results with the same level of detail as the other findings. The results presented are mostly qualitative, and, when quantitative measurements are performed, the variability within groups is often larger than the difference between groups. Thus, the interpretation of the results may be more interesting in the context of the disease severity; for example, were the patients with more neutrophil-platelet aggregates more likely to have complications than those with less. To overcome the issues due to the small cohort of patients enrolled in the study, the stratification of the patients based on disease severity, irrespective of the covid status may be more interesting. A larger study may also address other important issues, including the effect of neutrophil-platelet aggregation on 1. The activity of neutrophils, including their motility and 2. The activation of the platelets and the formation of small thrombi.

Is the work clearly and accurately presented and does it cite the current literature?

Yes

If applicable, is the statistical analysis and its interpretation appropriate?

Yes

Are all the source data underlying the results available to ensure full reproducibility?

Yes

Is the study design appropriate and is the work technically sound?

Yes

Are the conclusions drawn adequately supported by the results?

Partly

Are sufficient details of methods and analysis provided to allow replication by others?

Yes

Reviewer Expertise:

design of assays for neutrophil functional changes in patients

I confirm that I have read this submission and believe that I have an appropriate level of expertise to confirm that it is of an acceptable scientific standard, however I have significant reservations, as outlined above.

# Response

**Published:** 2021-05-12

**Authors:** Sarah Walmsley

**Affiliations:** University of Edinburgh, UK

We would like to thank the reviewer for their interest in our work.  We completely agree that this work is limited by the study size, but were keen to share our observations given the biological implications of some of our findings.  A longitudinal study would certainly be one way to delineate how neutrophil populations relate to disease progression and severity. The activation phenotype observed in COVID-ARDS and the formation of neutrophil-platelet aggregates we also find to be intriguing and absolutely agree that further work to delineate the effect of neutrophil-platelet aggregation on key neutrophil functions including their activity, motility and consequence for thrombus formation is worthy of further study.

# Peer review: Reviewer response for version 1

**DOI:** 10.21956/wellcomeopenres.18276.r43488

**Published:** 2021-05-10

**Authors:** Attila Mocsai

**Affiliations:** 1 Department of Physiology, Semmelweis University School of Medicine, Budapest, Hungary

In this manuscript, Reyes et al. test various aspects of neutrophil biology in COVID-19-related and non-COVID acute respiratory distress syndrome using an unbiased proteomic and metabolomic analysis of peripheral blood neutrophils, along with parallel samples of healthy control and moderate COVID-19 patients. The authors reveal that COVID-related ARDS is characterized by neutrophil signatures indicating Type I interferon responsiveness, neutrophil degranulation, neutrophil-platelet aggregates and metabolic changes.

 The authors' approach of proteomic and metabolic analyses provides important novel addition to the existing literature given that neutrophils are transcriptionally silent cells and are therefore underrepresented in gene expression (e. g. scRNASeq) studies. The experiments are appropriately designed and carefully conducted. Though the subject of the manuscript is of clear interest, the depth of the study is somewhat limited, given the small patient cohort sizes and the lack of further in-depth mechanistic studies (as mentioned by the authors at the end of the Discussion). This applies particularly to the analysis of dexamethasone treatment. Taken together, this is an important, although somewhat descriptive study on the possible contribution of neutrophils to COVID-19 pathogenesis.

 Specific comments:

1. Although the authors had four different groups (HC, NA, MC, CA; not mentioning CD here), they somewhat randomly select the results of which groups to show or not to show. This makes the study somewhat unbalanced. The authors should either show the results of all groups or provide a rationale for why one or more groups have been excluded from certain experiments.
2. To what extent may the observed metabolic changes be related to changes in neutrophil-platelet aggregation?
3. The authors should make a more definitive distinction between components of the platelet activation and coagulation cascades. Though they are related to each other, they are two mechanistically different aspects of hemostasis.
4. Fig 2C is a bit difficult to follow. The authors should consider showing the different samples in separate dot-plots.

Is the work clearly and accurately presented and does it cite the current literature?

Partly

If applicable, is the statistical analysis and its interpretation appropriate?

Yes

Are all the source data underlying the results available to ensure full reproducibility?

Yes

Is the study design appropriate and is the work technically sound?

Yes

Are the conclusions drawn adequately supported by the results?

Yes

Are sufficient details of methods and analysis provided to allow replication by others?

Yes

Reviewer Expertise:

neutrophil biology, inflammation

I confirm that I have read this submission and believe that I have an appropriate level of expertise to confirm that it is of an acceptable scientific standard, however I have significant reservations, as outlined above.

# Response

**Published:** 2021-05-10

**Authors:** Sarah Walmsley

**Affiliations:** University of Edinburgh, UK

We would like to thank the reviewer for their interest in our work and to address each of the points raised below.  We agree that the work is limited by sample size and that future mechanistic studies will be important moving forwards.

 Specific comments:

- Although the authors had four different groups (HC, NA, MC, CA; not mentioning CD here), they somewhat randomly select the results of which groups to show or not to show. This makes the study somewhat unbalanced. The authors should either show the results of all groups or provide a rationale for why one or more groups have been excluded from certain experiments.

We apologise for any lack of clarity as to the data sets shown.  Initial flow counts and surface phenotyping were shown for each of the disease / severity and dexamethasone treatment groups.  The focus of this program of work was to map the molecular basis for neutrophil reprogramming in the distinct clinical entities of COVID-19 ARDS and non-COVID ARDS, irrespective of different therapeutic strategies.  For this reason, further dissection of MC was not-undertaken and CA and CD were grouped for proteomic and metabolomic analysis.

  

- To what extent may the observed metabolic changes be related to changes in neutrophil-platelet aggregation?

It is interesting to question whether neutrophil-platelet aggregation results in a degree of metabolic reprogramming of neutrophils or vice versa.  The metabolic rewiring following neutrophil stimulation with type 1 IFNS in hypoxic cell culture, would appear in part to be independent of the formation of these aggregates as these experiments were conducted with healthy control blood neutrophils in platelet free culture conditions, verified by absence of CD41 (Figure 5C). 

  

- The authors should make a more definitive distinction between components of the platelet activation and coagulation cascades. Though they are related to each other, they are two mechanistically different aspects of hemostasis.

We apologise for any confusion caused.  The reason for retaining this grouping, was that as currently stands, we are unable to ascribe which of these proteins are platelet derived, and which are neutrophil derived with evidence that in murine models airspace recovered inflammatory neutrophils can themselves express some of these critical proteins (Watts et al.  JCI 2021).  The terms of reference used in figure 4 relate to specific GO terms enriched within the different disease groups. 

- Fig 2C is a bit difficult to follow. The authors should consider showing the different samples in separate dot-plots.

We apologise and now provide separate plots.

# Peer review: Reviewer response for version 1

**DOI:** 10.21956/wellcomeopenres.18276.r43077

**Published:** 2021-04-14

**Authors:** Véronique Witko-Sarsat

**Affiliations:** 1 Institut National de la Santé et de la Recherche Médicale U1016, Université Paris-Descartes, Paris, France

**General comments**

 This manuscript focuses on neutrophil state of activation in patients suffering from COVID-19-associated ARDS. Several reports have well demonstrated the key role of neutrophils during this condition but little is known about their functional disturbance.

 The aim of the study is to compare neutrophil populations in patients with COVID-19 ARDS to those of patients with non COVID-19 ARDS, moderate COVID and healthy controls. Of interest, the authors have studied the effect of dexamethasone on neutrophil parameters.

 Several aspects of neutrophil biology are covered including flow cytometry analysis of membrane marker phenotyping, proteomic and metabolomic analysis in a restricted number of patients which, as mentioned by the authors, represents a limiting aspect of the study. The methodology are carefully described. Overall the experiments reported have been well designed and performed. However, I have several issues and some potential suggestions that I would like to mention.

 This manuscript is a valuable study. It provides novel tracks of research on neutrophils in COVID-19 that will need to be investigated in more details to elucidate the underlying molecular mechanisms.

**1) Analysis of membrane markers in NDN and in LDN : relative importance of LDN**

 The authors have determined the neutrophil phenotype in normal density neutrophils (NDP) and in low density neutrophils (LDN) isolated from the PBMC fraction which are supposed to be immature neutrophils generated during emergency hematopoiesis. These LDN have been described in different inflammatory conditions.

 Although ARDS is associated with a severe neutrophil-dominated inflammation, the fraction of LDN in the blood of patients from the NA or the CA group is rather small since it is not significantly increased as compared to controls (in which basically no LDN could be detected). Surprisingly, the LDN fraction is increased after dexamethasone treatment in the CA group.

 The authors also observed that only a small fraction of LDN could be considered as immature neutrophils with a CD66bhigh-CD16low-CD10low phenotype.

 It might be useful to the reader to note that CD16 is not only a marker of maturity but could also be considered as a marker of activation or apoptosis.

**2) Activation state in different subtypes of neutrophils : LDN versus NDN versus whole blood analysis**

 In Figure 2, the authors observe that NDN do not display an activated phenotype since no modulation of CD62L or CD63 (calculated as fold change with controls) is observed. This conclusion is different in Figure 6K, in which the Facs data are expressed in MFI. This discrepancy requires clarification.

 In contrast, the authors show a decreased CD62L in LDN which is significant only in immature LDN CD66bhigh-CD16low-CD10-low in all types of patients.

 However, as I mentioned previously, this LDN fraction is rather small representing less than 2 to 5 % (although this is higher in CA around 10% without being significantly different from the other groups either NA and MC).

 If one consider that the population of CD66bhigh-CD16low-CD10-low could be involved in the pathogenesis of COVID-19 it might be relevant to quantify this population within whole blood.

 Maybe authors could show the data for each marker measured whithin whole blood in different groups with the percentage and the MFI.

 It could be important to question whether this non abundant sub-population of immature LDN could be physiologically relevant. **Taken together the role of LDN in the pathogenesis of ARDS in COVID-19 might not be prominent.  The authors might discuss this issue.**

**3) Analysis of dexamethasone effect**

 The authors show that dexamethasone treatment increases the percentage of mature LDN ( **Figure 2H**). It could be informative to have the number of treated patients.

 In each patient, does dexamethasone affect both the percentage and/or the maturity state of LDN?

 It would also be more informative to display the data for each patients to see if the decrease of immature LDN occurs in all the cases (instead of showing an histogram).

**4) Proteomic analysis of expression of coagulation-related proteins in neutrophils**

 The authors have observed several modifications of protein abundance using the label free mass spectrometry analysis.

 Although this analysis can give a quantitative view of each protein, the expression of specific proteins should be studied by Western blot analysis. It is not clear whether the mass spectrometry analysis would detect cleaved protein or whether only one isoform could be down or up-regulated.

 Western blot analysis is specially important for the proteins involved in fibrin clot formation which might be involved in the pathogenesis of COVID-19.

 Interestingly, the authors show that proteins involved in coagulation in neutrophils from dexamethasone-treated patients do not seem to be different from those of untreated patients when evaluated by proteomic analysis using the relative abundance.

 To strengthen this conclusion, it could be useful to do Western blot analysis of each patient (dexamethasone and untreated group should be analyzed) and to quantify the expression of this coagulation-related proteins with a control protein (actin or other non modulated protein). Dexamethasone might indeed modify the profil of these proteins by inducing post-translational modifications or cleavage in some of these proteins. This could be part of its potential anti-inflammatory activity.

**5) Expression of proteins involved in coagulation- expression of CD41 on neutrophils**

 In Figure 5C, the authors observe an increased expression of CD41 which is a marker of platelet activation. What are the mechanisms of CD41 expression on neutrophils? Is it a transfer of membrane from platelets or platelets or soluble CD41? Do activated platelets associate more easily on neutrophils from COVID patients?

 The authors show some modulation of proteins that can bind coagulation proteins. Figure 5E shows that CD11b is increased in NDN both in NDN and CA and this is evaluated by MFI values. This representation is difficult to compare with the data expressed in fold changes displayed in Figure 5F. Could the authors use the same method to express the results?

 Figure 5D shows that the expression of CD41 is significantly higher in NDN from mild COVID (MC) patients who do not experience ARDS or any thrombotic complications. In contrast, no increase in CD41 is observed in NDN from CA patients who are at risk of developing thrombotic complications.

 In addition, the authors show that CD41 is significantly increased in mature LDN both in MC and in CA. Since the proportion of mature LDN is not very important (less than 5% in MC and CA) in the neutrophil population, the pathophysiological relevance of this observation is questionable.

 It seems that variation of CD41 is not associated with the clinical state of COVID patients because it is observed in both MC and CA. Maybe the authors could comment on this.

 It is not mentioned (or no clearly enough) if the LDN from CA are mature or immature in Figure 5C.

 In the Figure 5, there is no panel showing the effect of resiquinod which activate TLR7 and TLR8 (although it is mentionned in the legend). In the text, the authors describe that this results in upregulation of CD11b and CD18. **The author should include the missing panel.**

**6) Evaluation of degranulation of neutrophils**

 As mentionned above, in Figure 6K, the authors report an increase in CD63 and CD66b membrane expression in CA whereas this increased is not significant on the data presented in Figure 2F expressed in fold changes. Are the same patients included? What should we conclude because there are two opposite comments after analysis of the same dataset?  The authors should clarify this discrepancy.

 In Figure 6N, the data depicting the effect of resiquinod on neutrophils from healthy controls under hypoxia are not shown although they are mentioned in the legend and in the text. Again, **the author should include the missing panel.**

 The panel 6L showing plasma concentration of MPO , lactoferrin and elastase would be more convincing with more than 3 patients.

**7) Metabolic analysis**

 The metabolic analysis and the observation of a disturbance in neutrophil metabolism in COVID patients is very interesting but it seems that some additional experiments would clarify the message.

 In Figure 8, the authors show that resiquinod can modify the metabolic profile of neutrophils from healthy controls by increasing glycolysis and this is decreased by type I IFN. What is the effect of TypeI IFN alone on glycolysis? Is glycolysis increased only by the combination resiquinod and type I IFN?

 The authors show that type I IFN decrease lactate under hypoxic conditions.

 Does that mean that glycolysis is decreased in the absence of activation of TLR7 and 8 by resiquinod? Could the authors show the analysis of glycolysis using the see horse technology to compare with the data shown in Figure 8A?

 In contrast, type I IFN did not affect the levels of TCA intermediates but increased the level of glutamate. Is the intracellular glucose affected?

 The metabolic analysis of neutrophils in Covid patients show that there is no modulation of lactate but an increase in intracellular glucose. This is why is could be important to measure intracellular glucose in the neutrophils of healthy controls stimulated by resiquinod in the presence or absence of type I IFN.

 Interestingly, the authors show an increase in glutamate levels in ARDS patients both in NA and in CA suggesting that this metabolic change is more related to an adaptation of neutrophils to ARDS but is not specific of COVID-19 ARDS.

 It could be relevant to have similar representation of glutamate amounts in the different experiments: in Figure 8I it is expressed in abundance (?) and in Figure 8D performed in healthy controls it is expressed in fold changes.

 To make the data consistent and get a clear picture of the metabolic changes that could occur in neutrophils from ARDS patients I would suggest to measure the glutamate abundance in neutrophils from heathy donors stimulated by either resiquinod in the presence or absence of type I IFN.

 Same suggestion applies to the analysis of glycolysis and glucose abundance.

**General comment:**

 Figures 5, 6 and 7 are very hard to understand because the size of the text is too small.  This should be modified in order to make this figure readable.

Is the work clearly and accurately presented and does it cite the current literature?

Yes

If applicable, is the statistical analysis and its interpretation appropriate?

Yes

Are all the source data underlying the results available to ensure full reproducibility?

Yes

Is the study design appropriate and is the work technically sound?

Yes

Are the conclusions drawn adequately supported by the results?

Partly

Are sufficient details of methods and analysis provided to allow replication by others?

Yes

Reviewer Expertise:

NA

I confirm that I have read this submission and believe that I have an appropriate level of expertise to confirm that it is of an acceptable scientific standard, however I have significant reservations, as outlined above.

# Response

**Published:** 2021-05-11

**Authors:** Sarah Walmsley

**Affiliations:** University of Edinburgh, UK

**General comments**

 This manuscript focuses on neutrophil state of activation in patients suffering from COVID-19-associated ARDS. Several reports have well demonstrated the key role of neutrophils during this condition but little is known about their functional disturbance.

 The aim of the study is to compare neutrophil populations in patients with COVID-19 ARDS to those of patients with non COVID-19 ARDS, moderate COVID and healthy controls. Of interest, the authors have studied the effect of dexamethasone on neutrophil parameters.

 Several aspects of neutrophil biology are covered including flow cytometry analysis of membrane marker phenotyping, proteomic and metabolomic analysis in a restricted number of patients which, as mentioned by the authors, represents a limiting aspect of the study. The methodology are carefully described. Overall the experiments reported have been well designed and performed. However, I have several issues and some potential suggestions that I would like to mention.

 This manuscript is a valuable study. It provides novel tracks of research on neutrophils in COVID-19 that will need to be investigated in more details to elucidate the underlying molecular mechanisms.

*We would like to thank the reviewer for their interest in our work and to address each of the points raised below.*

**1) Analysis of membrane markers in NDN and in LDN : relative importance of LDN**

 The authors have determined the neutrophil phenotype in normal density neutrophils (NDP) and in low density neutrophils (LDN) isolated from the PBMC fraction which are supposed to be immature neutrophils generated during emergency hematopoiesis. These LDN have been described in different inflammatory conditions.

 Although ARDS is associated with a severe neutrophil-dominated inflammation, the fraction of LDN in the blood of patients from the NA or the CA group is rather small since it is not significantly increased as compared to controls (in which basically no LDN could be detected). Surprisingly, the LDN fraction is increased after dexamethasone treatment in the CA group.

 The authors also observed that only a small fraction of LDN could be considered as immature neutrophils with a CD66bhigh-CD16low-CD10low phenotype.

 It might be useful to the reader to note that CD16 is not only a marker of maturity but could also be considered as a marker of activation or apoptosis.

*We agree that there are very low numbers of low-density neutrophils.  In NA LDN (immature and mature) account for 7 % of the total neutrophil population, which is further increased to 23 % considering all COVID-19 ARDS patients regardless of dexamethasone treatment.  This shift in neutrophil populations is further amplified by the increase in the absolute number of circulating neutrophils in patients with NA and CA, resulting in a significant increase in the absolute number of circulating LDN (new figure 2I).*

*We agree, it is of interest that dexamethasone treatment results in an increase in the LDN fraction in CA.*

*With respect to the markers of neutrophil maturity, we absolutely agree that CD16 has previously been reported as a marker of activation / apoptosis, hence our inclusion of an extra marker of maturity, CD10.  In this setting, CD16 expression parallels the expression of CD10 in circulating neutrophil populations.*

**

**2) Activation state in different subtypes of neutrophils : LDN versus NDN versus whole blood analysis**

 In Figure 2, the authors observe that NDN do not display an activated phenotype since no modulation of CD62L or CD63 (calculated as fold change with controls) is observed. This conclusion is different in Figure 6K, in which the Facs data are expressed in MFI. This discrepancy requires clarification.

 In contrast, the authors show a decreased CD62L in LDN which is significant only in immature LDN CD66bhigh-CD16low-CD10-low in all types of patients.

 However, as I mentioned previously, this LDN fraction is rather small representing less than 2 to 5 % (although this is higher in CA around 10% without being significantly different from the other groups either NA and MC).

*We apologise for any confusion caused.  In figure 2 D-F we are comparing expression between LDN and NDN, and the data provided is fold change to NDN populations within each disease state.  This analysis does not identify changes in NDN populations between disease groups, which is provided in figure 6K.  All raw data including MFI values have been provided as data uploads to accompany the manuscript. As detailed in response to point 1 above, LDN account for 23 % of the total PMN population considering all COVID-19 ARDS patients regardless of dexamethasone treatment, and a substantial increase in total numbers given their abundance in these patients (see new figure 2I).*

 If one consider that the population of CD66bhigh-CD16low-CD10-low could be involved in the pathogenesis of COVID-19 it might be relevant to quantify this population within whole blood.

 Maybe authors could show the data for each marker measured whithin whole blood in different groups with the percentage and the MFI.

*Please see new figure 2I above re total LDN counts.  Raw MFI values have been uploaded for every figure set within the materials that accompany this article.  We do not have MFI values for every surface marker used within the whole blood, as this staining was undertaken on LDN and NDN populations post percoll purification.  There are currently no specific surface markers that enable identification of LDN sub populations.*

 It could be important to question whether this non abundant sub-population of immature LDN could be physiologically relevant. **Taken together the role of LDN in the pathogenesis of ARDS in COVID-19 might not be prominent.  The authors might discuss this issue.**

*In light of the details provided above, we would argue that LDNs do significantly contribute to the circulating neutrophil pool.  We absolutely agree that future work is required to further dissect the importance of this sub-population of neutrophils to disease pathogenesis.*

**3) Analysis of dexamethasone effect**

 The authors show that dexamethasone treatment increases the percentage of mature LDN ( **Figure 2H**). It could be informative to have the number of treated patients.

*There were n=5 dexamethasone treated CA patients.*

 In each patient, does dexamethasone affect both the percentage and/or the maturity state of LDN?

*For clarification, we do not have paired data from the same individual pre and post dexamethasone.  Patients with CA received dexamethasone therapy once clinical criteria were met and prior to transfer to critical care for ventilatory support for ARDS.*

 It would also be more informative to display the data for each patients to see if the decrease of immature LDN occurs in all the cases (instead of showing an histogram).

*Please see comment above.*

**4) Proteomic analysis of expression of coagulation-related proteins in neutrophils**

 The authors have observed several modifications of protein abundance using the label free mass spectrometry analysis.

 Although this analysis can give a quantitative view of each protein, the expression of specific proteins should be studied by Western blot analysis. It is not clear whether the mass spectrometry analysis would detect cleaved protein or whether only one isoform could be down or up-regulated.

 Western blot analysis is specially important for the proteins involved in fibrin clot formation which might be involved in the pathogenesis of COVID-19.

 Interestingly, the authors show that proteins involved in coagulation in neutrophils from dexamethasone-treated patients do not seem to be different from those of untreated patients when evaluated by proteomic analysis using the relative abundance.

 To strengthen this conclusion, it could be useful to do Western blot analysis of each patient (dexamethasone and untreated group should be analyzed) and to quantify the expression of this coagulation-related proteins with a control protein (actin or other non modulated protein). Dexamethasone might indeed modify the profil of these proteins by inducing post-translational modifications or cleavage in some of these proteins. This could be part of its potential anti-inflammatory activity.

*It was not feasible with the limited number of neutrophils obtained from each patient to perform western blots for the proteins of interest identified.  We have tried, where possible, to validate functional changes using flow cytometry and immunofluorescence (IF).  Importantly, label free DIA mass spectrometry allowed us to quantify absolute protein abundance and provided a platform for unbiased analysis of the factors which differentiate healthy from CA and NA neutrophil populations.  We agree that it would be of interest to explore whether dexamethasone induces post translational modifications, but this falls out with the scope of this manuscript.*

**5) Expression of proteins involved in coagulation- expression of CD41 on neutrophils**

 In Figure 5C, the authors observe an increased expression of CD41 which is a marker of platelet activation. What are the mechanisms of CD41 expression on neutrophils? Is it a transfer of membrane from platelets or platelets or soluble CD41? Do activated platelets associate more easily on neutrophils from COVID patients?

*We do not have any direct evidence that neutrophils themselves are expressing CD41.  We do provide direct evidence of platelet neutrophil co-localisation (Figure 5C).  It is interesting to question whether activated platelets more readily co-localise with neutrophils.*

 The authors show some modulation of proteins that can bind coagulation proteins. Figure 5E shows that CD11b is increased in NDN both in NDN and CA and this is evaluated by MFI values. This representation is difficult to compare with the data expressed in fold changes displayed in Figure 5F. Could the authors use the same method to express the results?

*Apologies for any confusion caused.  Where in vitro culture of cells was undertaken, to allow for variability in mean fluorescent intensities (MFI) (normoxic unstimulated N-U) from different donors, we compared the effects of dexamethasone back to baseline untreated normoxic MFI values for each donor (Figure 5F).*

 Figure 5D shows that the expression of CD41 is significantly higher in NDN from mild COVID (MC) patients who do not experience ARDS or any thrombotic complications. In contrast, no increase in CD41 is observed in NDN from CA patients who are at risk of developing thrombotic complications.

 In addition, the authors show that CD41 is significantly increased in mature LDN both in MC and in CA. Since the proportion of mature LDN is not very important (less than 5% in MC and CA) in the neutrophil population, the pathophysiological relevance of this observation is questionable.

 It seems that variation of CD41 is not associated with the clinical state of COVID patients because it is observed in both MC and CA. Maybe the authors could comment on this.

*In light of the details provided above, we would argue that LDN do significantly contribute to the circulating neutrophil pool (23 %), with the absolute number of LDN substantially elevated in CA.  We agree that there is also increased expression in MC and consequently elevated CD41 is a feature of moderate to severe COVID-19 disease, but not ARDS all cause.  Future work exploring expression in mild and asymptomatic disease would be of interest in this regard.*

 It is not mentioned (or no clearly enough) if the LDN from CA are mature or immature in Figure 5C.

*We have been unable to successfully undertake CD16 IF due to technical limitations. Nevertheless, we postulate that the neutrophils stained in Figure 5C are mature neutrophils given their multilobulated nuclear appearance, a characteristic feature of mature neutrophils.*

 In the Figure 5, there is no panel showing the effect of resiquinod which activate TLR7 and TLR8 (although it is mentionned in the legend). In the text, the authors describe that this results in upregulation of CD11b and CD18. **The author should include the missing panel.**

*Apologies for any confusion caused. For clarification, data in Figure 5F are neutrophils treated in hypoxia with resiquimod in the absence or presence of increasing doses of dexamethasone compared to an untreated hypoxic control (H-U).*

**6) Evaluation of degranulation of neutrophils**

 As mentionned above, in Figure 6K, the authors report an increase in CD63 and CD66b membrane expression in CA whereas this increased is not significant on the data presented in Figure 2F expressed in fold changes. Are the same patients included? What should we conclude because there are two opposite comments after analysis of the same dataset?  The authors should clarify this discrepancy.

*Apologies for any confusion caused. The data presented in Figure 2F and Figure 6K are of the same patients. As detailed above, in Figure 2F, we are comparing expression between LDN and NDN, and the data provided is fold change to NDN populations within each disease state.  This analysis does not identify changes in NDN populations between disease groups, which is provided in Figure 6K.*

 In Figure 6N, the data depicting the effect of resiquinod on neutrophils from healthy controls under hypoxia are not shown although they are mentioned in the legend and in the text. Again, **the author should include the missing panel.**

*Apologies for any confusion caused. Data in Figure 6N are neutrophils treated in hypoxia with resiquimod (H-R) or without resiquimod (H-U), fold-changed to a normoxic untreated control.*

 The panel 6L showing plasma concentration of MPO , lactoferrin and elastase would be more convincing with more than 3 patients.

*As highlighted in our discussion, we acknowledge the relatively small number of patients as a limitation of our study and thus agree that an increase in n number would strengthen our conclusions.*

**7) Metabolic analysis**

 The metabolic analysis and the observation of a disturbance in neutrophil metabolism in COVID patients is very interesting but it seems that some additional experiments would clarify the message.

 In Figure 8, the authors show that resiquinod can modify the metabolic profile of neutrophils from healthy controls by increasing glycolysis and this is decreased by type I IFN. What is the effect of TypeI IFN alone on glycolysis? Is glycolysis increased only by the combination resiquinod and type I IFN?

*In initial experiments under normoxic conditions, IFN alone does not modify glycolytic uplift following activation with fMLP as measured by seahorse.  For the manuscript, we used TLR activation with resiquimod to parallel physiological states in COVID-19 disease and in light of this preliminary work and the limited number of seahorse wells, we did not include type 1IFN alone.*

 The authors show that type I IFN decrease lactate under hypoxic conditions.

 Does that mean that glycolysis is decreased in the absence of activation of TLR7 and 8 by resiquinod? Could the authors show the analysis of glycolysis using the see horse technology to compare with the data shown in Figure 8A?

 In contrast, type I IFN did not affect the levels of TCA intermediates but increased the level of glutamate. Is the intracellular glucose affected?

*LC-MS analysis was undertaken in the absence of TLR7 and 8 activation, to address the basal metabolic response to neutrophil stimulation with Type 1 IFNs.  It will be important in future work to address the metabolic rewiring that occurs in response to TLR7 and 8 activation in the presence and absence of Type 1 IFNs.*

*Unfortunately, due to potential risk of infection from blood taken from patients with COVID-19 disease, we were unable to perform live seahorse assays in this setting.*

*Levels of glucose -6-phosphate were unchanged.*

 The metabolic analysis of neutrophils in Covid patients show that there is no modulation of lactate but an increase in intracellular glucose. This is why is could be important to measure intracellular glucose in the neutrophils of healthy controls stimulated by resiquinod in the presence or absence of type I IFN.

*As detailed above, we have not undertaken LC-MS analysis of resiquimod stimulated neutrophils.  We agree this would be an important future direction of work, and a limitation of the current study.  This is reflected in the cautious interpretation of our data as presented.*

 Interestingly, the authors show an increase in glutamate levels in ARDS patients both in NA and in CA suggesting that this metabolic change is more related to an adaptation of neutrophils to ARDS but is not specific of COVID-19 ARDS.

*We agree.*

 It could be relevant to have similar representation of glutamate amounts in the different experiments: in Figure 8I it is expressed in abundance (?) and in Figure 8D performed in healthy controls it is expressed in fold changes.

*We apologise, for this inconsistency.  We have now replaced figure 8 B-D with absolute abundance (AU)*

 To make the data consistent and get a clear picture of the metabolic changes that could occur in neutrophils from ARDS patients I would suggest to measure the glutamate abundance in neutrophils from heathy donors stimulated by either resiquinod in the presence or absence of type I IFN.

*We agree this would be interesting to explore further, but to expedite the publication of neutrophil disease phenotype, we feel that this currently lies out-with the scope of the current program of work.*

 Same suggestion applies to the analysis of glycolysis and glucose abundance.

*We agree, please see comment above.*

# Peer review: Reviewer response for version 1

**DOI:** 10.21956/wellcomeopenres.18276.r42926

**Published:** 2021-04-01

**Authors:** Lynne Prince, Rebecca Dowey

**Affiliations:** 1 Department of Infection, Immunity and Cardiovascular Disease, University of Sheffield, Sheffield, UK

This study by Reyes et al describes a comprehensive molecular analysis of neutrophils isolated from people with ARDS. They find distinct neutrophil activation signatures in COVID-19 ARDS, perhaps not surprisingly, centred around anti-viral IFN responses. The study also sheds light on the association of COVID-19 with vascular thrombosis, by identifying evidence of platelet neutrophil binding and activation of the clotting cascade. Overall, this work has been performed to a very high standard, is beautifully written and given the significant mortality associated with ARDS of all aetiology, it is of great clinical importance.

 We have the following questions:

1. Could the authors clarify and add detail to the neutrophil isolation method - is this a plasma/percoll gradient as per the Haslett reference cited, or it a modified protocol?
2. For figure 2B, were equivalent numbers of events acquired for each dot plot? This would give a more accurate indication of the proportion of each cell type.
3. Considering the overarching aim of the study is to compare COVID-19 ARDS vs non-COVID-19 ARDS, it is surprising to not see a direct comparison between CA and NA for figure 3E and figure 4A. Was this done and what was the outcome?
4. In figure 5C, were the authors able to visualise neutrophil:platelet aggregates for more than one cell and could this quantified and data presented? This would add support to figure 5D.
5. For figure 6 (M-N), consider performing stats on the raw data values and not fold changes.
6. The legend for figure 5F and figure 6N suggests that normoxic conditions (N-U) were studied but this does not seem to be reflected on the figure. Could this be clarified?

Is the work clearly and accurately presented and does it cite the current literature?

Yes

If applicable, is the statistical analysis and its interpretation appropriate?

Yes

Are all the source data underlying the results available to ensure full reproducibility?

Yes

Is the study design appropriate and is the work technically sound?

Yes

Are the conclusions drawn adequately supported by the results?

Yes

Are sufficient details of methods and analysis provided to allow replication by others?

Yes

Reviewer Expertise:

Neutrophil biology, inflammation, cell survival

We confirm that we have read this submission and believe that we have an appropriate level of expertise to confirm that it is of an acceptable scientific standard.

# Response

**Published:** 2021-04-09

**Authors:** Sarah Walmsley

**Affiliations:** University of Edinburgh, UK

We would like to thank the reviewers for this positive feedback, we address each of the points below.

**1. Could the authors clarify and add detail to the neutrophil isolation method - is this a plasma/percoll gradient as per the Haslett reference cited, or it a modified protocol?  **Apologies for any confusion.  To clarify, we are using a modified PBS/percoll gradient (Dransfield et al., Cell Death and Disease. 2015; 6:E1646).  Cell pellets are resuspended in 50% percoll.  This cell suspension overlays a 73% lower phase and 63% upper phase discontinuous percoll gradient.  Normal density neutrophils are recovered from the interface between the lower 2 phases, and low density neutrophils and peripheral blood mononuclear cells are recovered from the interface between the upper 2 phases following centrifugation (720 g, break 0, acceleration 1 for 20 mins).

**2. For figure 2B, were equivalent numbers of events acquired for each dot plot? This would give a more accurate indication of the proportion of each cell type.  **For each dot plot we acquired data on a fixed volume of whole blood (100 microlitres) to allow for differences within multiple different circulating cell populations. 

**3. Considering the overarching aim of the study is to compare COVID-19 ARDS vs non-COVID-19 ARDS, it is surprising to not see a direct comparison between CA and NA for figure 3E and figure 4A. Was this done and what was the outcome?  **This is an important point.  We compared the neutrophil proteome of each disease group back to the healthy control neutrophil proteome to allow us to identify which protein signatures were common to both COVID-19 ARDS and non-COVID-19 ARDS, and which were specific to each disease group.  A direct comparison between COVID-19 ARDS and non-COVID-19 ARDS would not allow us to identify changes in protein expression that were common to ARDS all cause versus healthy control. 

**4. In figure 5C, were the authors able to visualise neutrophil:platelet aggregates for more than one cell and could this quantified and data presented? This would add support to figure 5D. **Direct visualisation of neutrophil:platelet aggregates was undertaken in low density neutrophil fractions following density centrifugation and FACS.  Given the relatively small cell yield, we did not feel it appropriate to quantify this imaging modality. 

**5. For figure 6 (M-N), consider performing stats on the raw data values and not fold changes.  **We are not claiming a difference in neutrophil granule release following dexamethasone treatment in COVID-19 ARDS patients (Figure 6M).  To allow for variability in mean fluorescent intensities (MFI) in healthy donor populations at baseline (normoxic unstimulated N-U), we compared the effects of hypoxia and resiquimod back to baseline untreated MFI values for each donor (Figure 6N).

**6. The legend for figure 5F and figure 6N suggests that normoxic conditions (N-U) were studied but this does not seem to be reflected on the figure. Could this be clarified?  **Apologies for any confusion.  As detailed above, each sample was compared back to the normoxic unstimulated control (N-U) to calculate the fold change, hence its inclusion in the figure legend.
